# Scaling laws: legal and social complexity in US localities

**DOI:** 10.1098/rsta.2023.0151

**Published:** 2024-04-15

**Authors:** Elliott Ash, Christoph Goessmann, Suresh Naidu

**Affiliations:** ^1^ ETH Zurich, Zurich, Switzerland; ^2^ Columbia University, New York, NY, USA

**Keywords:** legal complexity, city complexity, scaling laws

## Abstract

Law sets out the rules for society and the economy, particularly important for interactions between strangers. Legal code is a form of non-rival infrastructure, a public good important for investment and innovation. This paper investigates whether legal code complexity scales with population size in US localities. We analyse a corpus of municipal codes from 3259 cities and measure legal complexity using various metrics, including number of words, bytes, and compressed bytes. We find that legal complexity scales geometrically with jurisdiction population, with a scaling parameter of approximately 0.2 and an $R^{2}$ of approximately 0.2. The estimated scaling parameter is similar to gross domestic product *per* *capita*, consistent with an interpretation of legal codes as regulating social interactions *per capita* in cities.

This article is part of the theme issue ‘A complexity science approach to law and governance’.

## 1. Introduction

Law plays a crucial role in society by setting out the rules for interactions between individuals. This is particularly important for interactions between strangers, where there is no pre-existing relationship or trust. The legal code, which is embedded in language and written down as a set of rules, serves as a form of infrastructure that provides a framework for economic and social activities (e.g. [1]). It is important for investment and innovation, as it provides certainty and predictability for individuals and businesses.

Previous research in quantitative urban studies has shown that a variety of variables scale with city size. For example, Bettencourt *et al.* [2] found that variables measuring output, such as gross domestic product (GDP), patents and wages, scale with city size at a reliable superlinear rate. They found that these variables follow a power law relationship, with an exponent of approximately 1.2. Similarly, variables measuring the volume of infrastructure, such as miles of roads, also scale with city size, but sublinearly, with an exponent of approximately 0.8. A ‘physics of cities’ approach to urban processes provides elegant and satisfying formal models that generate these numbers as strong predictions.

In this paper, we extend this approach to legal code complexity. Does legal code scale with population size? Is legal code more like output (superlinear) or more like infrastructure (sublinear)? We empirically analyse this question using a new corpus of municipal codes from 3259 US cities matched to information on local population. We measure legal complexity using tools from information theory [3]. The preferred measure is the file size (in bytes) of the compressed municipal codes.

We show that legal code, like the other city variables, is strongly correlated with population size. The length of the legal code extends sublinearly with city size, with a coefficient of about 0.2. In supporting results, we show that compressed size and uncompressed size scale with population at almost identical rates. That is, the law does not become more or less textually efficient as it grows, and our scaling relationships do not reflect redundancies in the legal code or ‘boilerplate’. Correspondingly, we estimate similar coefficients when using other measures of the volume of legal code, such as the word count.

Next, we look at the coefficients for some alternative text-based sophistication measures, motivated by the hypothesis that law is an optimal encoding of types of conflicts, so that the average ‘codeword’ is longer in larger cities. Overall, increasing population is associated with more complex sophisticated law, as measured by word length, sentence length and the diversity of the vocabulary. These coefficients are much smaller and less stable, and perhaps reflect that we do not have a theoretically grounded measure of law as an optimal code.

Finally, we measure the relationship between the length of the (compressed) legal code and population in two other contexts. We have information on the text of new legislation enacted by US state government, and the text of judicial opinions published by state appellate courts. We show that these measures on introduction of new laws over time scale with state population at a higher rate (between 0.4 and 0.7) than the rate for city population and the stock of law, but still sublinear, so consistent with infrastructure rather than economic output. We also have information on the text-based complexity of collective bargaining agreements, which we match to ‘number of covered employees’ as a corresponding measure of population. Again, we find a strong linear relationship with a sublinear coefficient.

We conclude the paper with a discussion about the estimated magnitudes. Given that it is sublinear, legal code might appear more like infrastructure than output. But this is misleading, because of the non-rival nature of law. The same law regulates all instances of a given type of conflict, and types of conflict are proportional to social interactions *per capita*. Legal code should scale like GDP *per capita*, i.e. social interactions divided by population. The positive relationship between legal complexity and population size can be explained by the need to regulate arm’s-length interactions between individuals in larger cities. As the population increases, the social distance between individuals also increases, leading to a greater need for legal rules and regulations, but at a decreasing rate.

Still, there are many open questions about the mechanisms underlying the relationship between population and legal code size. For example, it could be due to the volume or diversity of interpersonal conflicts, or by the volume or diversity of economic activity. Those are interesting questions for future work.

## 2. Background

### (a) Scaling laws in city size

The scaling laws observed in city size have been extensively studied in the interdisciplinary field on the ‘physics of cities’, arising out of urban economics and other quantitative social sciences analysing cities. One of the classic findings is Zipf’s Law, which states that the size distribution of cities follows a power law relationship.

**Figure 1.**
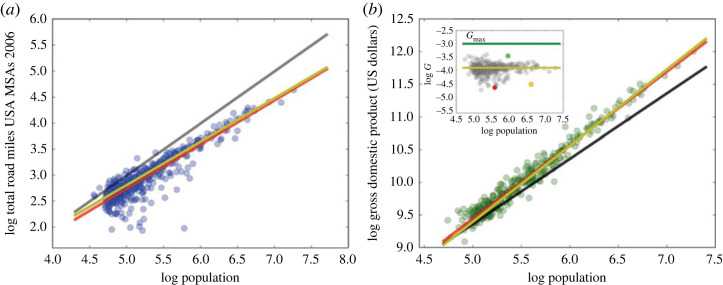
Scaling laws in cities. Source: Bettencourt [4]. (Online version in colour.)

In addition to Zipf’s Law, Bettencourt *et al.* [2] found that many variables scale with city size. That is, for some city outcome Y, we have $Y=aN^{\beta }$, where N is city population and β is the scaling factor (figure 1).

The previous work has focused on two categories of variables describing cities: those measuring productivity and those measuring the volume of infrastructure. For variables measuring productivity, such as GDP, patents and wages, Bettencourt *et al.* [2] found that they scale superlinearly with city size. These variables follow a power law relationship, with an exponent of β≈1.2. For variables measuring the volume of infrastructure, such as miles of roads, there is also a power law relationship, but it is sublinear—an exponent β≈0.8. This means that as city size increases, the volume of infrastructure increases, but at a slower rate compared to variables measuring productivity.

The scaling laws observed in city size can be explained by a basic trade-off between agglomeration and congestion. As cities grow, there are increasing agglomeration benefits, such as access to a larger labour market and knowledge spillovers. However, there are also increasing congestion costs, such as traffic congestion and higher housing prices. The balance between these benefits and costs determines the optimal size of a city.

### (b) Explanations for scaling: physics

The scaling laws observed in city size can be explained by both physical and economic factors. A physical model is provided by Bettencourt [4], who proposed a mechanistic explanation based on the geometry of interactions in a circular city. According to this framework, the local productive interactions in a city, which can be measured by income, are proportional to the number of pairwise interactions per unit area. The local productive interactions generate income that is proportional to the infrastructure costs of transporting every individual across the diameter of the city. Thus we obtain a relationship where area scales with population with a coefficient of $\beta =\frac{2}{3}$, and so transportation infrastructure (which are linear in physical distance) scale with coefficient $\frac{1}{3}$ and income, proportional to population squared over area, scales with coefficient $\frac{4}{3}$.

While simple, intuitive and roughly correct, this model does not have quite the right scaling coefficient, which is 1.2 for the superlinear case and 0.8 for the sublinear case. As we detail in the electronic supplementary material, appendix, Bettencourt [4] augments the model above by having individual interactions occur via decentralized networks, and so the mean distance between individuals is proportional to the inverse square root of population density: more people per unit area means the average distance between them falls, but less than proportionally, as would be the case, for example, in small-worlds networks where the average network distance is a logarithmic function of the total population. Further, Bettencourt [4] has GDP proportional to the number of interactions per total network area. These two assumptions yield a superlinear scaling parameter of GDP with population of 7/6 and a sublinear scaling of infrastructure with population with coefficient 5/6. GDP *per capita*, being driven by social interactions per total network area *per capita*, scales with parameter 1/6, as does the volume of social interactions *per capita*.

Some more formal details are presented in electronic supplementary material, appendix C.

### (c) What about law?

Law is made of language, which itself displays a variety of scaling laws [5]. But beyond complex and robust communication between individuals, legal codes also regulate disputes, both between individuals, as well as between individuals, organizations and governments (e.g. [6]). In addition, law serves a pivotal coordinating function, setting expectations and allowing coordinated and efficient transactions [7]. We can think of a volume of social interactions, which generate a proportional number of types of potential conflicts and coordination problems that need to be resolved by the legal system. For smaller communities and less complex societies, informal norms suffice (e.g. [8]). But at some point in increasing social complexity, those conflict management rules and coordinating norms become encoded as legal text (e.g. [9]). Once resolved and encoded into law, that type of dispute or dilemma can be more readily adjudicated in every future instance. Put differently, we can say that laws are non-rival in effect once written down.

At the level of the individual, the probability of any given conflict or coordination problem will increase with the number of social interactions—that is, social interactions *per capita*. If legal code works to efficiently encode this increasing variety of social conflicts and dilemmas, we would expect the legal code to increase in the number of interactions *per capita*.

As cities increase in population, the number of interactions increases. That is partly due to increased population density and more local interactions on the street. A person in a larger city also has more opportunities to physically meet with more people and businesses, as they are easier to get to. Further, cities tend to have more non-physical interactions through remote and digital communications.

As in the discussion above of the Bettencourt [4] model, due to the organization of social and physical networks, local social interactions do not scale quadratically with population, but instead scale with parameter 7/6. Taking the assumption that the legal code increases in interactions *per capita* with the rate at which interactions increase with population together, the volume of legal text might scale with number of interactions *per capita*, i.e. 7/6−1=1/6. As we will see this is remarkably close to the coefficient we observe in the data.

Of course, not all laws work to regulate pairwise interactions. Take rules on taxes, for example, which are about the relationship between a taxpayer and government. Or rules on government administration, which are only about interactions within government organizations. This model of social interactions does not apply to those types of provisions. Still, in these cases, one would expect that rules around these things would have to increase as more potential conflicts or unusual actions might be observed.

Further, many entities that are focal points for law, such as corporations, are not physically embodied and do not take up space. A city could have thousands of corporations that are providing digital services, requiring many legal provisions, but without any connection to physical proximity or population density.

### (d) Explanations for scaling: economics

In economics, Mulligan & Shleifer [1] provide an alternative model for how laws might scale with city size. In their model, laws regulate conflicts, and there is some frequency distribution f(L) across the different types of conflicts, ordered such that higher L means less frequent conflicts. They assume a constant benefit b per person of writing a law to cover a conflict and a fixed cost of writing a law k. Based on these assumptions, the legislators should write laws until the marginal benefit is equal to the fixed cost,

2.1 $$bNf(L^{*})=k,$$

which gives the equilibrium legal stock $L^{*}$. So long as there is a decreasing density f, a monotonic relationship between law and population will be observed. If f(⋅) is a power law, then $L^{*}\propto N^{\beta }$ with β>0. But the model does not generate predictions on the magnitude of β.

### (e) Municipal codes

To investigate the scaling laws of legal code complexity, we take as our main application municipal codes in US cities. Municipal codes are the collection of laws and ordinances that govern specific cities or towns. Each city has its own set of codes that are often tailored to its unique needs or circumstances. The codes cover a broad range of topics, including regulations on land use, zoning and building codes. The ordinances are also related to public health and safety, such as sanitation, noise control and animal regulations. Additionally, municipal codes regulate local business activities, manage public resources like parks and roads, and set guidelines for local government administration and elections. These codes are regularly updated to reflect new developments and changing needs within the community.

## 3. Methods

### (a) Data collection and pre-processing

This paper uses a corpus of municipal codes from 3259 US cities. We obtained the raw municipal codes from LexisNexis. These are the compiled sets of effective statutes and do not include rules from local administrative bodies, for example utilities or school boards. We removed markup to keep only the ordinance texts. We also de-duplicated the data by keeping the most recent version of each clause. More detail on the data processing is included in electronic supplementary material, appendix A.

**Figure 2.**
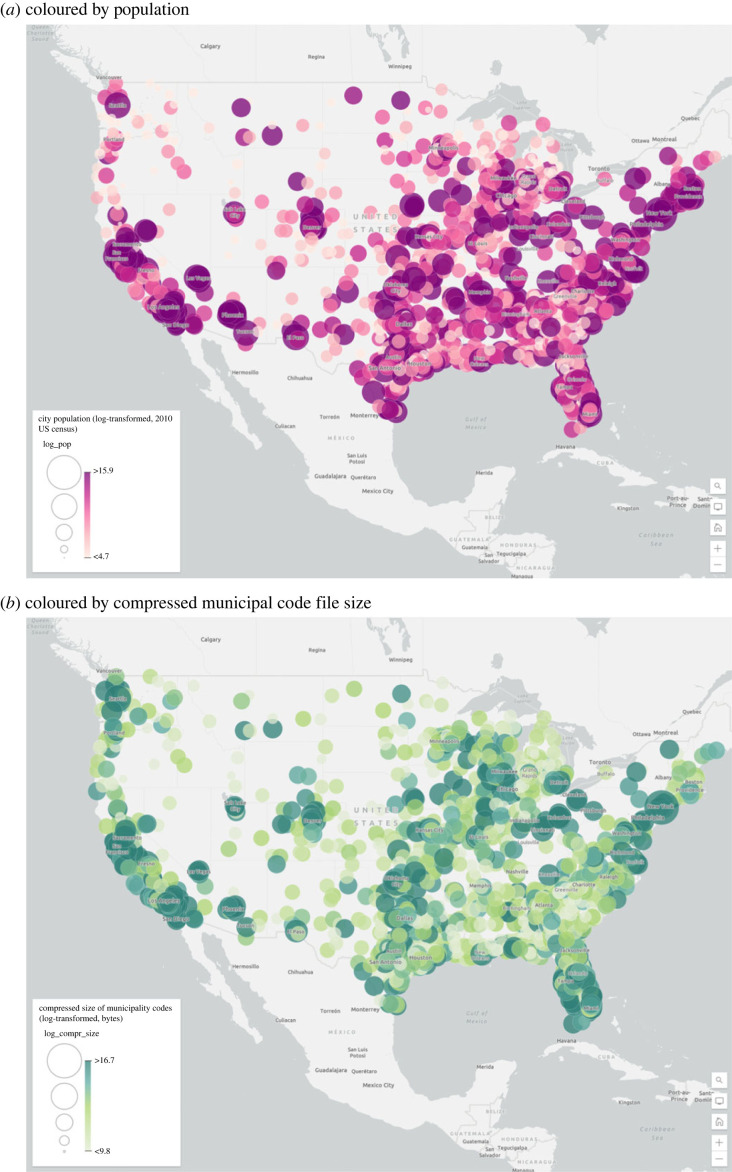
Geography of the dataset. A map showing the cities in our dataset, coloured by rank of population (*a*) and by compressed municipal code file size (*b*). (Online version in colour.)

We have data on city variables, notably population, from the US census. Our core empirical results consist of correlations between a variety of summary statistics of the text of the municipal codes and the logarithm of population, following the scaling literature discussed above (figure 2).

In the supporting results on legal scaling in alternative jurisdictions, we use information on the texts of state legislation and state court opinions collected by Ash *et al.* [10]. Data on collective bargaining agreements in Canada come from Ash *et al.* [11], while those from Brazil come from Lagos [12].

### (b) Complexity measures

We measured legal complexity from text, following recent work in legal linguistics [3,13]. We use various complexity metrics, including the number of words, bytes and compressed bytes in the legal code. We also calculated linguistic sophistication measures, such as characters per word and words per sentence, as well as entropy measures, such as word entropy.

**Figure 3.**
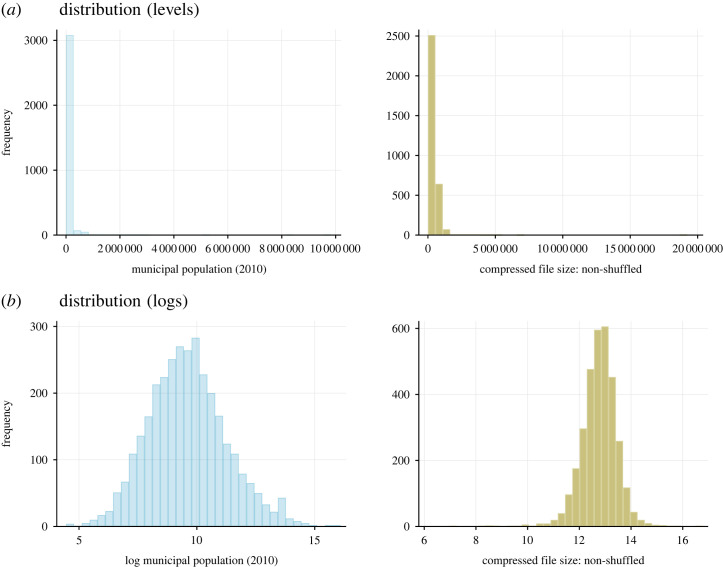
Distributions of city population and municipal code size. Information on the distributions of city populations (left plots) and the compressed file size of municipal code texts (right plots). Includes histograms in levels (*a*) and histograms in logs (*b*). (Online version in colour.)

Table 1 presents summary statistics of the various text-based measures of legal complexity. Table 2 shows pairwise correlations between the measures. As can be seen in figure 3, both city population and municipal code size are heavily skewed. For each, the log of the variable (figure 3*b*) is much closer to a normal distribution than the level (figure 3*a*), already suggesting some thicker-than-exponential tails.

**Table 1.** Summary statistics.

variable (logs)	N	mean	s.d.	min.	max.
city population	3243	9.61	1.66	4.45	16.10
uncompressed size (bytes)	3259	14.00	0.69	7.68	18.16
compressed size (bytes)	3259	12.77	0.69	6.97	16.77
compressed/uncompressed ratio	3259	1.23	0.03	0.71	1.75
word count	3259	11.78	0.73	7.10	15.57
sentence count	3259	9.05	0.69	3.04	13.01
section count	3259	7.03	0.73	2.20	10.04
characters per word	3259	1.62	0.02	1.56	1.83
word per sentence	3259	3.13	0.12	2.57	5.82
text entropy	3259	1.49	0.01	1.47	1.65
unique words used	3259	8.92	0.32	5.09	10.19

**Table 2.** Correlation matrix across text complexity measures.

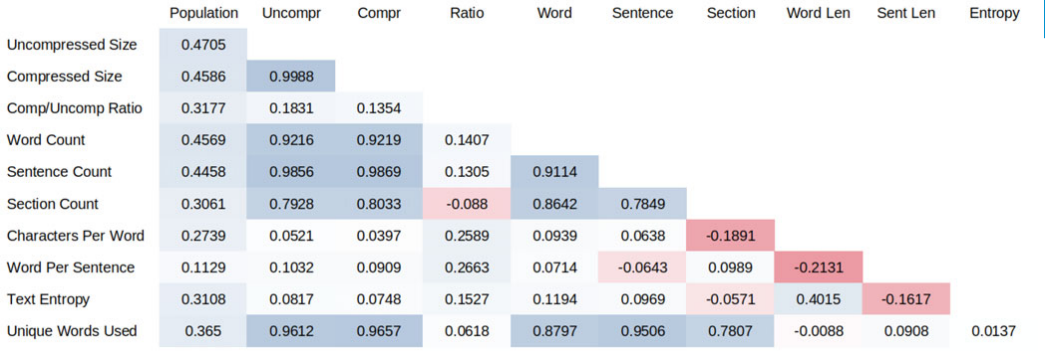

## 4. Results

### (a) Scaling of legal complexity with population

**Figure 4.**
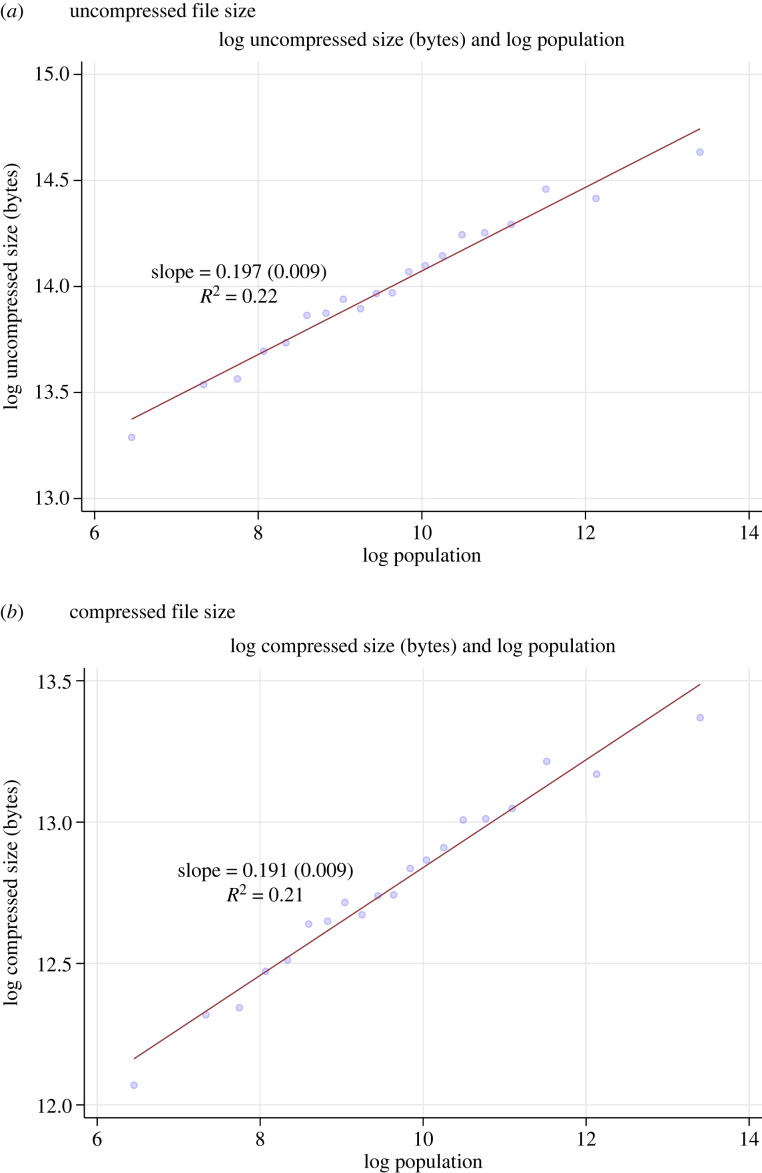
Municipal code size and city population. Binned scatter plots of legal complexity measure (vertical axis) and log city population (horizontal axis). (*a*) Vertical axis is log uncompressed file size in bytes. (*b*) Vertical axis is log compressed file size in bytes. Statistics give OLS slope coefficient, standard error in parentheses and $R^{2}$. (Online versionin colour.)

Figure 4 illustrates the relationship between municipal code size and city size with binned scatter plots. Figure 4*a* shows the relationship between log municipal code size (in uncompressed bytes) and log city population. Figure 4*b* shows the same but with compressed code file size. We can see that there is a strong positive relationship between code size and city size. The coefficient estimates and $R^{2}$ are the same regardless of compression. Legal complexity increases with population size and the relationship is linear on the log–log scale, suggesting a power law relationship between legal complexity and population. Code increases with population at a rate of about β=0.2. The $R^{2}$ is quite high at 0.2, meaning that city population explains substantial cross-city variation in legal code size.

Some supporting results are reported in the electronic supplementary material, appendix. First, electronic supplementary material, appendix figure A.1, shows the relationship between compressed file size and city population but as a scatter plot, rather than a binned scatter plot, showing each observation on the full vertical scale. The strong positive and linear relationship is still crystal clear.

Second, electronic supplementary material, appendix figure A.3, shows the plot for city population and the ratio of compressed to uncompressed file size. Given that the slopes for each of these variables on their own are so similar we expect a small coefficient here. While significant and positive, it is small, at 0.006. This means that codes do not tend to be come more repetitive, or use more boilerplate, as they get longer with population size. One interpretation of this finding is that legal code is kept relatively efficient at all scales in our sample. If anything, larger cities have a higher information content in their legal code compared to smaller cities.

Bettencourt [4] reports a range of 0–0.25 as the empirical bounds on the parameter governing how social interactions *per capita* scale with population size, where recall that the theoretical prediction was $\beta =\frac{1}{6}$. Our estimate is very close to the theoretical prediction, and well within the empirical range, consistent with our model linking the volume of law to the volume of social interactions in the city.

The physics-based theoretical model of legal scaling was motivated by the frequency of pairwise social interactions. That is, as cities become larger, populations become more dense, and more interactions and conflicts occur. To get at this theoretical mechanism more directly, we also relate legal complexity to population density (population per square mile). As shown in electronic supplementary material, appendix figure A.3 panel A, there is also a very strong relationship between complexity and density, with a similar $R^{2}$ and larger coefficient of β=0.3. That statistical relationship even holds when we control for city population (electronic supplementary material, appendix figure A.3 panel B), and the $R^{2}$ doubles to 0.44, suggesting that there is an additional need for more laws when there is higher density, over and above the total number of people covered by laws.

### (b) Alternative measures of text complexity

To assess the robustness of the relationship on city size and code size, we produce statistics using some alternative measures of legal code size and complexity. Electronic supplementary material, appendix figure A.4, reports similar binned scatter plots and slope statistics for three alternative legal detail measures: counts over words, sentences and sections. If fine-grained details about how legal concepts are written and distributed across documents are responsible for our results, then we should expect to see differences in how these alternative measures of legal code size vary with population. First, and most notably, the statistics using log word count are identical to those with file size: the log–log slope is β=0.2 and the $R^{2}$ is 0.21. Sentence count is also nearly identical with β=0.19, $R^{2}=0.2$. Section count is not as close at β=0.14, $R^{2}=0.09$, probably reflecting formatting and drafting style differences in how sections are split up across cities. The results for word count and sentence count give us additional confidence in the robustness of these relationships. Further, they give us confidence in using log word counts as a legal complexity measure when file sizes are not available (as below).

**Figure 5.**
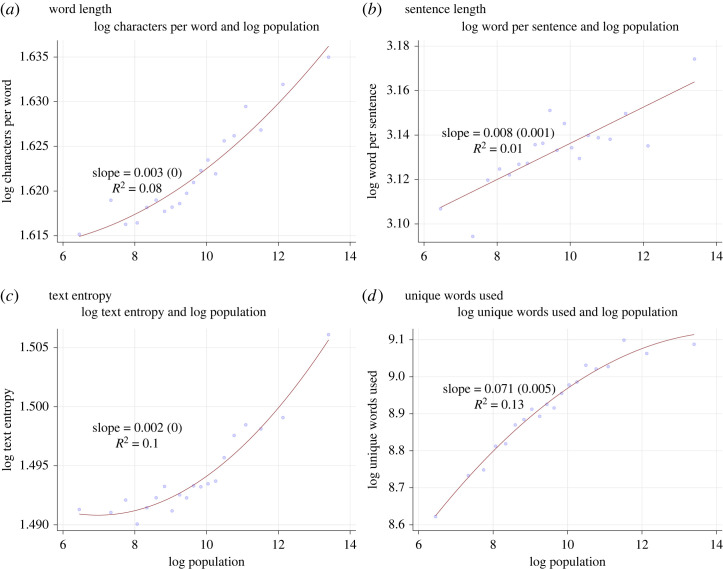
Readability and text entropy measures. How city population (horizontal axis) is related to readability or sophistication measures in municipal codes: word length (*a*), sentence length (*b*), entropy in the frequency distribution over words (*c*) and the number of unique words used (*d*). All variables are in logs. Statistics report OLS slope coefficients, standard errors and $R^{2}$. (Online version in colour.)

Next, we show the relationship between population size and some alternative text-based complexity measures that reflect linguistic sophistication or readability. Figure 5 shows that word length (figure 5*a*), sentence length (figure 5*b*), text entropy (figure 5*c*) and vocabulary size (figure 5*d*) are all positively increasing in city size, but none show the 0.2 linear scaling documented above for volume of text. For all of these, the slope is relatively small and $R^{2}$ is low. So text sophistication does not necessarily proxy for legal volume. Still, in general, we observe positive relationships between these complexity measures and population size, indicating that legal complexity tends to increase with population size across different dimensions.

### (c) Comparison with other corpora

To further validate our findings, we compared the scaling of legal complexity with population size to other corpora. First, we use texts from US state governments as an alternative local government legal text source. Here, we have information on newly enacted statutes over time, rather than a recent snapshot of the compiled code. Those are taken from Ash *et al.* [10], who collected from HeinOnline. Further, we have the collection of judicial opinions published in state appellate courts over time. That is available from Ash & MacLeod [14], who assembled the opinions from Bloomberg Law and LexisNexis. We can match the time series of legal documents to historical state population for the years 1900–2010.

This comparison differs to the city analysis in three ways. First, as producing compressed file sizes for these historical corpora is somewhat complicated, we use the log word count as the preferred measure of the volume of legal output. Second, since it is panel data, we cluster standard errors by state to allow for serial correlation across years within state. Third, we use time fixed effects to isolate cross-sectional comparisons and report ‘within’ $R^{2}$ that summarizes explained variation net of the fixed effects.

**Figure 6.**
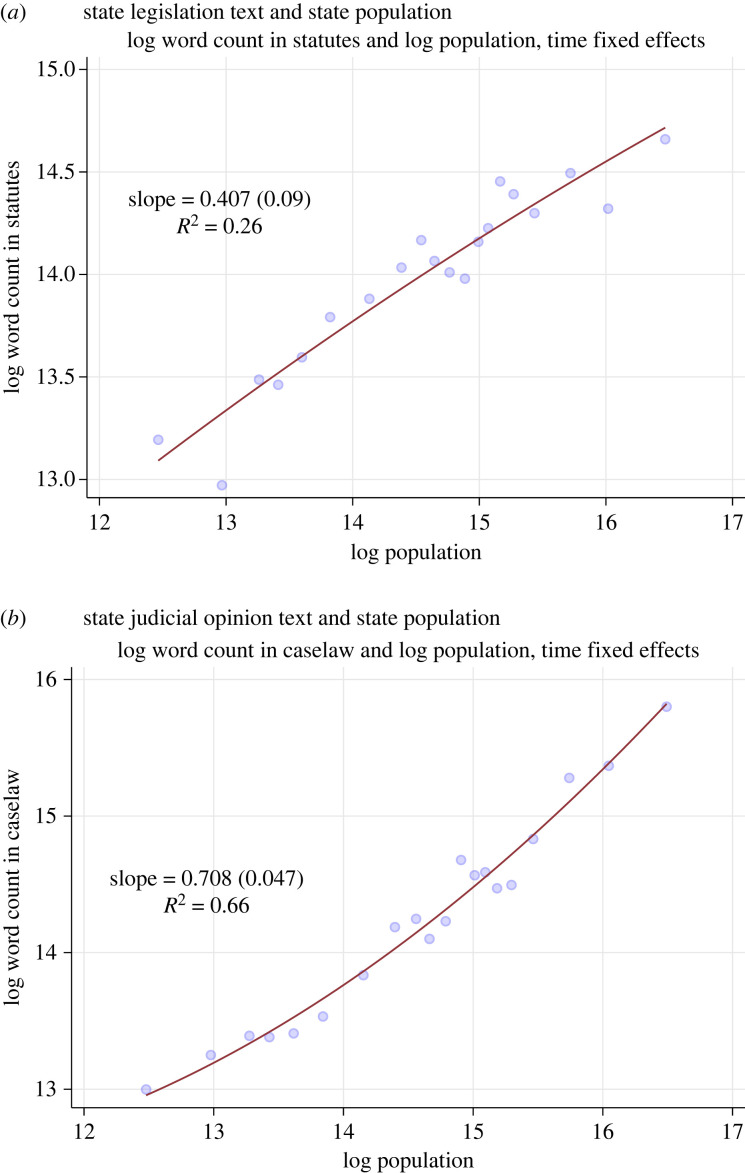
Scaling results for volume of state legislation and judicial opinions. How state population (horizontal axis) is related to the number of words in state legislation (*a*) and judicial opinions (*b*) published by year. All variables are in logs. Statistics report OLS slope coefficients, standard errors (clustered by state) and $R^{2}$ (within $R^{2}$, meaning the variation explained after residualizing out fixed effects). Time fixed effects absorbed. (Online version in colour.)

Figure 6 shows the results for how current population is associated with current volume of text in statutes and legal opinions published in a year. As with the overall size of city codes, state code size substantially reflects state population. In fact, it is an even stronger relationship than with the cities. Legislation increases with population at a rate of β=0.4 with $R^{2}=0.26$; judicial opinions correspondingly increase at a rate of β=0.7 with $R^{2}=0.66$.

An advantage of the panel nature of the state data is that we can look at changes in these metrics over time. Electronic supplementary material, appendix figures A.5 and A.6, show analogous plots and results for legislation and courts, respectively, but for 20-year periods since 1900. We can see that there is a substantial linear relationship throughout the time period, which has become somewhat stronger over time. For legislation, we see β=0.25 in the earliest period, increasing to β=0.54 in the latest period. For courts, it is more stable, with β=0.7 in the earliest period and β=0.78 in the latest period.

Further, we can look at within-state changes in population and published legal text. Electronic supplementary material, appendix figure A.7, shows that after taking out state fixed effects, there is still a positive relationship. That means that as a state’s relative population increases, so does its relative legal publication intensity. And the slope is still quite substantial, with β=0.13 for legislation and β=0.44 for court opinions. This means that if a state’s population increases by 10% in a given year, there is predicted to be a 1.3% increase in enacted legislative texts, and a 4.4% increase in published judicial opinion texts.

Finally, as an alternative source of legal text besides government-produced laws, we look at a private legal ordering as codified in labour union contracts. For Canada and Brazil, available from Ash *et al.* [11] and Lagos [12], respectively, we have large corpora of contracts matched to metadata on the company, namely the number of employees covered as an analogue to city or state population. In Brazil, the level of coverage is typically a sector by local labour market, while in Canada the level of coverage is typically an establishment or firm.

**Figure 7.**
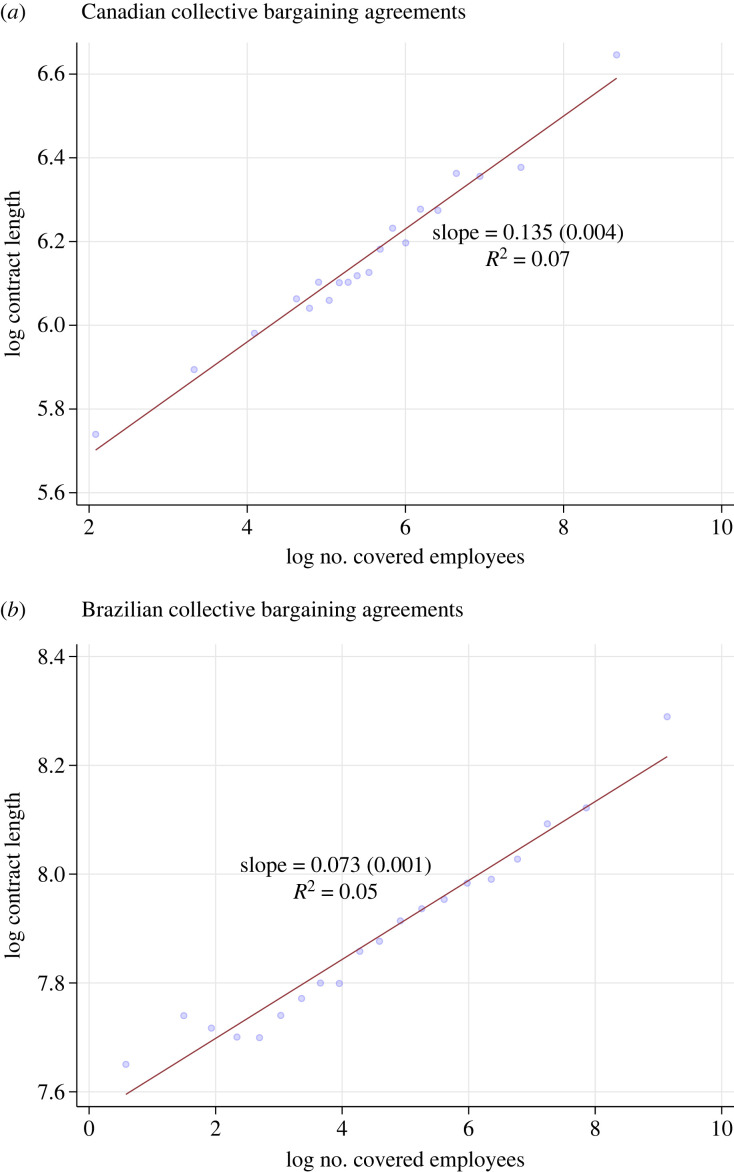
Collective bargaining agreements: length versus employees. How company size measured by number of employees population (horizontal axis) is related to the number of words in collective bargaining agreements, separately for Canada (*a*) and Brazil (*b*). All variables are in logs. Statistics report OLS slope coefficients, standard errors and $R^{2}$. (Online version in colour.)

These plots and estimates are shown in figure 7. In both Canada (figure 7*a*) and Brazil (figure 7*b*), there is a clear and strong positive linear relationship. However, it is less tightly connected than for the government-produced laws. The estimated rates of increase are β=0.14 for Canada and β=0.07 for Brazil, with $R^{2}$, respectively, at 0.07 and 0.05. Union collective bargaining agreements are negotiated between unions and employers (or employer associations) and are constrained by concerns of employer profitability as well as the outside options of unionized workers. The fact that the roughly log-linear relationship between jurisdiction size (here bargaining unit) and amount of text governing the jurisdiction remains in the very different context of private orderings in the labour market is an intriguing suggestion that the regulation–population relationships we have uncovered at the political level above is revealing of a more general mechanism.

## 5. Discussion

This work has documented a robust positive relationship between the volume of law regulating a jurisdiction and the number of people in the covered jurisdiction. This work builds on previous literature on scaling laws in cities, which previously has focused on how productivity and physical infrastructure scale with respect to city size [4]. We find that legal code volume scales strongly and sublinearly with the number of people in the covered jurisdiction, with a scaling parameter of approximately 0.2 in the cross section for municipal code size and city size.

Our simple social interaction-based model of legal volume would predict $\beta =\frac{1}{6}$, which is not far from the estimated $\beta =\frac{1}{5}$. Taking that model at face value, we can say that a positive relationship between legal complexity and population size can be explained by the need to regulate social interactions between individuals in larger cities. As the population increases, the number of interactions between individuals also increases, leading to a greater need for legal rules and regulations. This is reflected in the increase in the absolute amount of law, which scales with population size. The 0.2 parameter is remarkably close to the 1/6 parameter on the volume of social interactions in Bettencourt [4] and well within the range of empirical estimates given in that paper.

A statistically significant positive sublinear relationship, albeit with different parameter values, holds across a range of jurisdictional contexts—US cities, US state legislatures, US state courts, Canadian union contracts and Brazilian union contracts—and across different dimensions of the complexity of legal code—compressed or uncompressed text file size, word/sentence counts and text sophistication scores.

Law adapts to the needs of a growing population. It provides a framework for economic and social activities, ensuring that interactions between individuals are regulated and conflicts are resolved. As population size increases, the legal code becomes more complex, reflecting the increasing complexity of interactions between strangers. This complexity provides certainty and predictability for individuals and businesses, enabling them to make informed decisions and engage in economic activities.

On the other hand, the estimated growth rates are not constant across contexts. Looking at the publication of new laws in the state legislatures, we estimate a higher rate of β=0.4, and an even higher rate in state courts of β=0.7. In the labour union contracts, we estimate lower rates of β=0.07--0.14.

There could be many reasons for these differences. For example, is it intuitive that court opinions are more sensitive to population, as they are more directly connected to the number of conflicts in a jurisdiction. Teasing out such mechanisms is an important area for future work. For example, one could look at a wider range of covariates and evaluate their influence with multivariate regressions. Or one could look at differences in scaling across different legal topics, such as laws regulating social interactions versus laws regulating public goods.

In this vein, it will be an interesting area for future work to consider other dimensions of complexity. For example, one could use the structure—i.e. sequential structuring—of the codes. The number of sections, or the depth or hierarchy of clauses, would be informative on complexity and specialization. Similarly, one could measure cross-references within the code. One could then understand whether increases in linguistic volume are also associated with more branching and speciation of topics. Developing such specialized measures would be quite valuable to better understand the scope of these patterns.

Such alternative complexity measures could draw even more from information theory. An efficient coding perspective on law in our framework could imply that the average ‘legal codeword’ is proportional to the negative entropy of types of social conflict. If social conflicts are uniformly distributed across interactions, then $E[\mathrm{codeword}]=\frac{1}{6}\log(N)$. We lack a well-defined measure of legal codewords, and for the proxies for codewords we have examined here, we do not see this relationship and leave further examination to future work.

Such extensions would require significant data collection and development of causal research designs that are beyond the scope of this paper. While there may be diverse proximate mechanisms behind these differences in scaling parameters, the uniformly sublinear scaling also suggests some general forces may be at play, and we leave further investigation of this to subsequent work.

## Data Availability

Replication data are published on Harvard DataVerse. Supplementary material is available online [15].
